# ‘FlexYonio’; a reliable instrument to support monitoring the length of the long finger flexors in Duchenne muscular dystrophy (DMD)

**DOI:** 10.1177/22143602251362027

**Published:** 2025-08-17

**Authors:** Saskia LS Houwen- van Opstal, Maaike Pelsma, Yolanda MEM van den Elzen, Laura JB Merkenhof, Tamara P Popping- De Vries, Jan T Groothuis, Menno van der Holst, Edith HC Cup

**Affiliations:** 1 Department of Rehabilitation, Donders Institute for Brain, Cognition and Behaviour, Radboud University Medical Center, Amalia Children's Hospital, Nijmegen, The Netherlands; 2 Department of Rehabilitation, Donders Institute for Brain, Cognition and Behaviour, Radboud University Medical Center, Nijmegen, The Netherlands; 3 Department of Orthopedics, Rehabilitation and Physiotherapy, Leiden University Medical Center, Leiden, the Netherlands

**Keywords:** Duchenne muscular dystrophy, reliability, long finger flexors, outcome measure

## Abstract

**Background:**

Shortening of the long finger flexors (FDP) results in extension limitation of both wrist and fingers and can hinder important activities of the upper extremities in Duchenne muscular dystrophy (DMD). Early detection of FDP shortening is important for timely interventions, but reliable measurement of FDP length is difficult.

**Aim:**

To develop a new instrument to monitor FDP shortening more easily and precisely and to determine its intra -and interrater reliability.

**Materials and methods:**

The new instrument, called the ‘FlexYonio’, was clinically developed according to biomechanical standards to be able to monitor FDP length, by measuring range of motion of wrist extension with extended fingers. A prospective reliability study was conducted during annual outpatient visits of people with DMD at the neuromuscular center of the Radboudumc. Repeated measures were conducted and two raters assessed the FDP length with the new instrument using a standardized measurement protocol. Reliability was measured using intra class correlation coefficient (ICC) calculation.

**Results:**

The ‘FlexYonio’ was easy to use and became part of the standard daily clinical care in DMD in the Radboudumc. For reliability, 86 arm/hands were assessed; the intra- and interrater reliability were excellent with ICC > 0.99. The within-rater limits of agreement were −6 to 8 degrees and the between-raters −11 to 13 degrees.

**Conclusion:**

The ‘FlexYonio’, in combination with the standardized measurement protocol, is a promising, easy to use, and reliable tool to support the monitoring of FDP shortening in people with DMD, who are able to extend their fingers.

## Introduction

Duchenne muscular dystrophy (DMD) is an x-linked, progressive neuromuscular disease, that occurs 1 in 5000 live-born boys.^1^ Disease course typically starts with proximal muscle weakness in the early stages of the disease, leading to loss of ambulation around 8–14 years, and more distal weakness, also affecting arm and hand function in the later disease stages. The life expectancy of people with DMD is increasing far beyond their twenties due to corticosteroids, scoliosis surgery, the use of mechanical ventilation, together with good clinical practice.^2,3^ People with DMD depend on their arm function for the largest part of their lives. Unfortunately, their arm function also declines, and with this also the ability to perform activities with their upper extremities decreases.^4^ Besides the decline of arm function, also shortening of the muscles occurs in people with DMD leading to contractures of the joints.^2^ Shortening of the long finger flexors (flexors digitorum profundus, FDP) is common in DMD, especially during the late non-ambulatory disease stage.^5^ Shortened FDP result in a decreased ability to extend the wrist and fingers. Wrist extension is crucial as it provides optimal positioning for hand use and table top activities.^6^ It is important to monitor the length of the FDP, in order to be able to intervene before functional abilities are limited.

In absence of golden standards to monitor the length of the FDP, the most accepted way to indicate FDP length is to measure the extension of the wrist with extended fingers.^7^ In practice, this is done manually with help of a goniometer. However, because of the long trajectory of the FDP over multiple joints, it can be hard to confine small flexion movements in the interphalangeal joints of the fingers while measuring the wrist extension in clinical practice. Currently there is no tool which enables a reliable measurement of FDP length counteracting finger flexion while extending the wrist. Therefore this study aims to develop a tool based on biomechanical standards to adequately measure wrist extension with extended fingers, indicating FDP length. Secondly, this study investigates the intra- and interrater reliability of this tool enabling better and more timely treatment planning.

## Methods

### Development of FlexYonio

**Figure 1.**
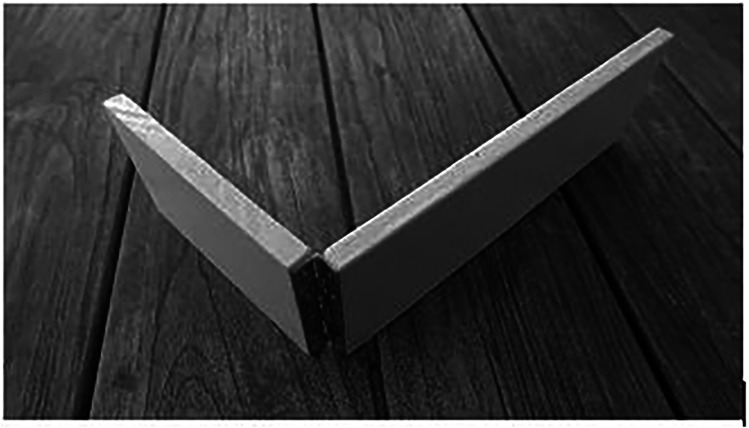
The ‘FlexYonio’ exists of two wooden boards, brought together with a hinge.

**Figure 2.**
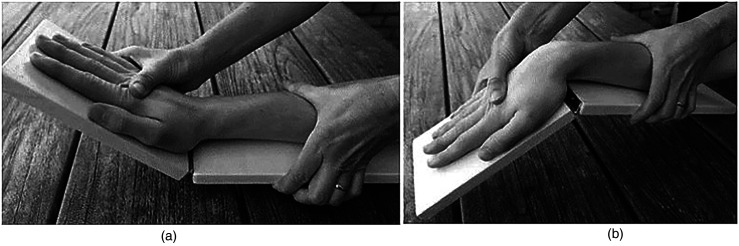
Positioning of the “FlexYonio”. 2A. “FlexYonio” position if wrist extension > 0. 2B. “FlexYonio position if wrist extension < 0. Figure 2A shows a situation in which the patient is able to extent the wrist with extended fingers. Figure 2B shows the situation when extension of the wrist with extended fingers is below the neutral position, in this case the ‘FlexYonio’ can be reversed.

To minimize inaccuracies during assessment of the length of the FDP the ‘FlexYonio’ has been developed within the neuromuscular team by an occupational therapist of the Radboud university medical center (Radboudumc)(YvdE) (see Figure 1). The ‘FlexYonio’ exists of two wooden boards, brought together with a hinge, which is positioned at the wrist joint. The design of the ‘FlexYonio’ enables measurement with extension of the wrist, taking into account all joints and muscles needed (Figure 2A). In case a limitation of the wrist to neutral or flexed position exist due to extension limitation or contracture the FlexYonio can be used in inverted position. (Figure 2B).

The ‘FlexYonio’ is used in clinical practice by our neuromuscular team since 2016 to determine the presence of shortening of the FDP. The ‘FlexYonio’ helps to stabilize the fingers in extension and it is easily seen if the distal phalanges tend to flex during the extension of the wrist.

### Measurement protocol

To standardize the measurements, two examinators are needed, one to hold the ‘FlexYonio’ in position and the other to measure with a digital goniometer. The position of the examiners was defined in a measurement protocol which can be found in the Supplementary Material. For the intra-rater reliability, the measurement was repeated three times by the same rater on both sides. For interrater reliability, the set of measurements (three times on both sites) was repeated one hour later by a second rater who was blinded to the outcomes of the first measurements. The raters were trained occupational and physical therapists from our neuromuscular team.

### Design and participants

A prospective study was conducted between September 2020 and November 2021. Males above 12 years old, with genetically confirmed DMD, who visited the outpatient clinic of the neuromuscular center of the Radboudumc were approached by mail to participate, two weeks before their visit. For inclusion, participants had to be able to passively fully extent the metacarpophalangeal joints, proximal interphalangeal joints, and distal interphalangeal joints of all fingers. Each participant and / or their parents provided written informed consent.

### Data management

An independent researcher collected the measurement outcomes and entered them in Castor EDC database. Data were anonymized and handled according to the guidelines of good clinical practice. This study was approved by the local medical ethical committee of the Radboudumc (No. 2020-6996).

### Statistical analyses

The data was checked for normal distribution. For intra-rater reliability the intra-class correlation coefficient (ICC) was calculated based on single measurements (3 repeated measurements), absolute agreement, two-way mixed-effects model.^8^ For the interrater reliability, ICC was calculated based on a mean rating (2 raters), absolute agreement, two-way random-effects model.

The ICC was classified as follows: poor <0.50, moderate 0.50–0.74, good 0.75–0.90, and excellent >0.90.^8^ Based on the guidelines for health measurement validation studies, the sample size for the interrater reliability study is 50.^5^ Bland-Altman plots were used to examine the limits of agreement (mean +/- 1.96 SD) of the differences between raters and within raters (comparing the differences between the first and last measurement).^9,10^ The standard error of the mean (SEM) was calculated over all measurements. Because no gold standard exists for this measurement, the predetermined minimum sample size was estimated on n = 50, which is derived from the formula of Giraudeau and Mary, in which the desired ICC was stated on 0,8 and CI 95%.^11^

All data was analyzed using SPSS version 25.0 (IBM SPSS, Inc., Armonk, New York).

## Results

**Figure 3.**
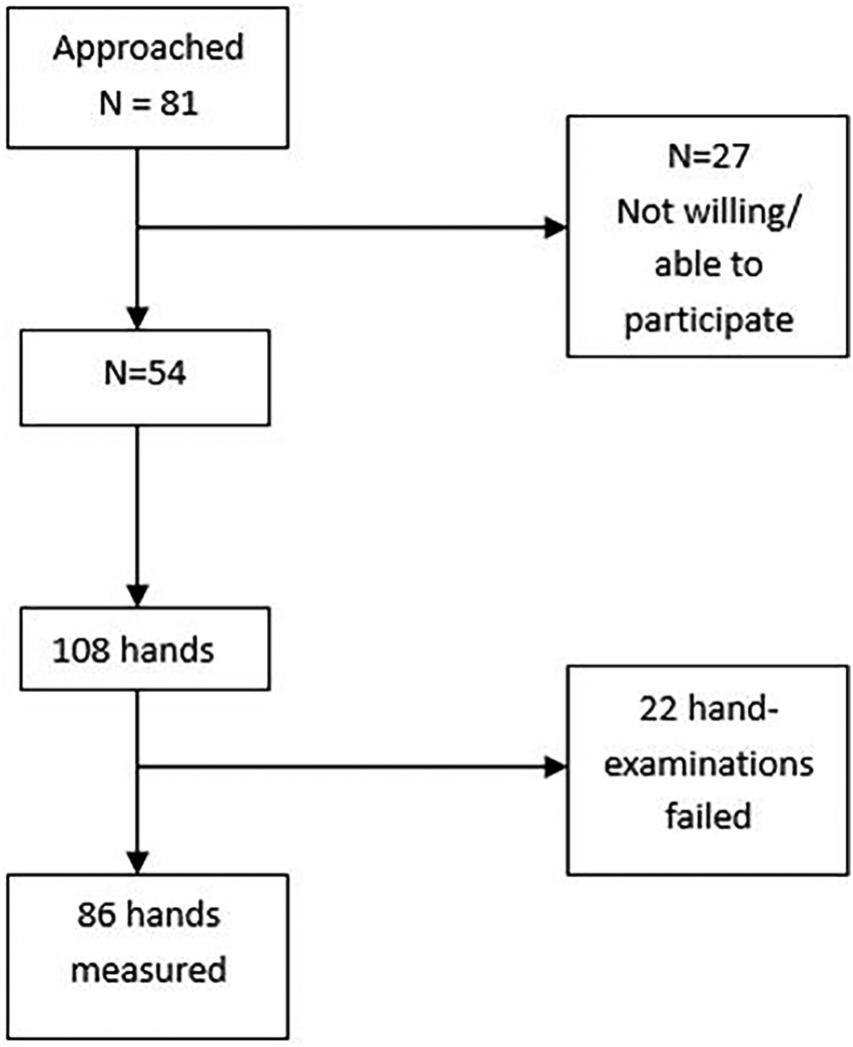
Inclusion FlexYonio study.

Of the 81 males with DMD who were approached, 54 were willing and able to participate; some outpatient visits were canceled because of COVID-19 related restrictions. Of the 54 patients, 108 arm/ hands were measured, 22 measurements failed because full extension of the finger joints was not possible or too painful. For analyses, 86 arm/ hand-measurements of 44 patients with DMD were included (see Figure 3). The mean age of the participants was 20 years old (range 12–42 years).

**Figure 4.**
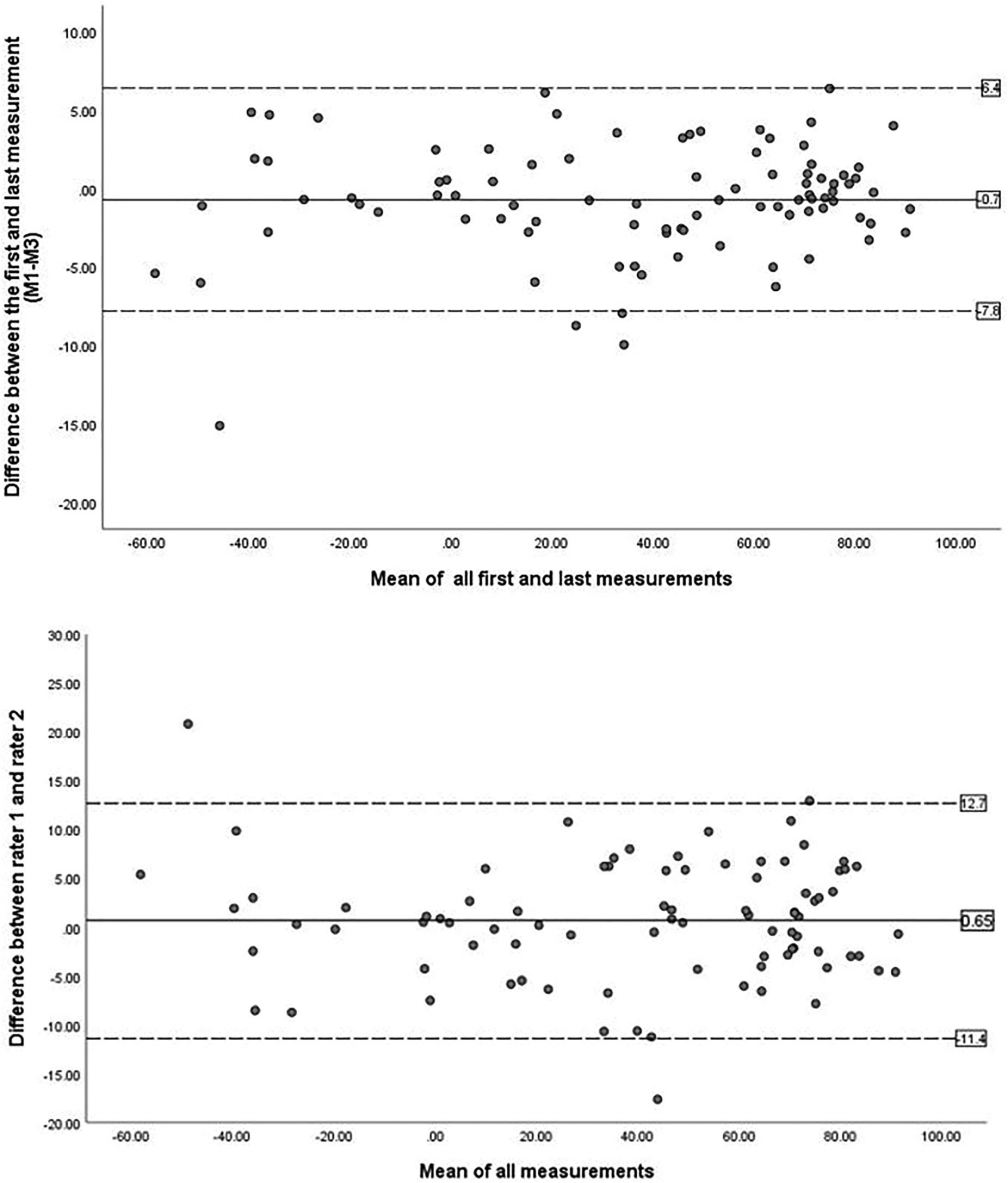
Blant-Altman plots; 4A. Intra-rater reliability (M1-M3). 4B. Interrater reliability (R1-R2). In both figures, the X-axis represents the average of the measurements and the Y-axis represents the differences between the measurements. The midline is representing the mean differences between the measurements. 95% of the values (confidence interval) falls within the upper and lower bound (limits of agreement) which is represented by the dotted lines.

Most patients had the ability to extend the wrist with extended fingers, leading to a skewed normal distribution of all measurements. However, the differences between the measurements of the raters and between the raters were normally distributed. The intra- and interrater reliability of the ‘FlexYonio’ was excellent with ICC above 0.99 (see Table 1). The limits of agreement of the intra-rater reliability were maximal −8 to 6 degrees, which means that 95% of the differences between measurements within one rater was smaller than 8 degrees. The limits of agreement of the measurements between the raters (interrater) was −11.4 to 12.7 degrees (see the Blant-Altman plots, Figures 4A and B). Descriptive data of all comparisons, including the mean difference, standard deviations, and limits of agreement are presented in Table 2. The mean difference between measurements within and between raters was 0.7. The SEM of all measurements was 4.6 degrees.

**Table 1.** Reliability analysis (absolute agreement, 2 raters, 3 measurements per rater).

	Intra-rater reliability ICC (95% CI)	Inter-rater reliability ICC (95% CI)
*Rater 1*	1.00 (0.99, 1.00)	0.99 (0.99, 1.00)
*Rater 2*	0.99 (0.99, 1.00)	

**Table 2.** Bland Altman descriptive statistics.

	Mean difference	1.96*SD	Limits of agreement^b^
R1; Comparing M1 and M2	−0.59	8.03	−9° to 7°
R1; Comparing M2 and M3	−0.22	6.47	−7° to 6°
R1; Comparing M1 and M3	−0.81	7.05	−8° to 6°
R2; Comparing M1 and M2	−0.24	8.44	−9° to 8°
R2; Comparing M2 and M3	−0.28	6.24	−7° to 6°
R2; Comparing M1 and M3	−0.53	9.54	−10° to 9°
Comparing mean R1 and R2	0.65	12.00	−11° to 13°

Abbreviations: R1/2, rater 1/2; M1/2/3, measurement 1/2/3; SD, standard deviation.

^b^ Limits of agreement: mean difference ± 1.96*SD (CI 95%).

## Discussion

With the new developed ‘FlexYonio’ and its measurement protocol, the difficulties of fixating the wrist and finger joints at the same time disappear and more accurate monitoring of FDP length becomes possible. The ‘FlexYonio’ is easy to build, with no need for expensive materials, and therefore suitable for any practice.

The development of the ‘FlexYonio’ derived from a clinical need to be able to better fixate the wrist and finger joints. Clinically, we already experienced the benefits, this study underscribes the use of the ‘FlexYonio’ in clinical practice. Early detection of shortening of the FDP is paramount, as the longitudinal progression is not linear, but can decrease dramatically, especially in Brooke 4.^5^ Preventive measures are needed before this timeframe to preserve muscle length, which is important in a variety of activities.^6,12^ As soon as contractures exist, it is hardly possible to reverse this process.^13^ We recommend that the presence of contractures should always be investigated, registered and taken into account.

The current study shows an excellent intra- and interrater reliability of this new developed ‘FlexYonio’ for measuring the extension of the wrist with extended fingers, which represents the length of the FDP. In general, for goniometric measurements a maximum measurement error of 10 degrees is acceptable.^9^ In the current study > 95% of the repeated measurements of one rater was within this range. Between the raters, in 92.5% of the measurements there was a difference of less than 10 degrees. Larger difference between measurements could appear due to several options: because of different pressure applied on the fingers during repeated measures or between raters, the position of the hinge of the ‘FlexYonio’ related to the wrist joint, or degree of relaxation of a person with DMD at the moment of the measurement. No structural differences were seen in measurements which were done at the beginning of the outpatient consultation and at the end, which means that this was not a factor of influence in reliability.

We learned that measuring FDP length is not always possible, especially in progressed disease staged, then the ‘FlexYonio’ could not be used. This was in case of contractures in the finger joints, or when moving the fingers is too painful. The latter occurred in people with DMD being in their late non-ambulatory phase (Brooke >4), and in people with DMD who were sensitive or had anxiety for pain.

Some strengths and limitations have to be considered while interpreting the results of this study. The strength of this study is that the measurements were done in a standardized manner, which can be implemented in any other setting. Besides, a great variety of people with DMD in different disease stages were measured. An important limitation is that when an ulnar deviation contracture of the wrist was presents, the ‘FlexYonio’ measurement might have been influenced. The high ICC scores suggest that this is not of influence on the reliability, however, in clinical practice, it is an important factor to include in upper limb analyses. A second limitation is that the ‘FlexYonio’ has not been compared to traditional manual testing with the goniometer of the wrist extension with extended fingers. In our clinical practice, the manual method is not used anymore, because of the difficulties fixating the fingers during the measurements. For this reason, we see the ‘FlexYonio’ as an addition to the manual testing, more than an alternative. Manual testing is still useful to assess joint mobility of the wrist and fingers in case full extension of all finger joints at the same time is not possible at all (independent of the position of the wrist) and the FlexYonio cannot be used, for example in more progressed disease phases. We have experienced that the ‘FlexYonio’ can be of great help to fixate the fingers for measuring the (limited) wrist extension with extended fingers. This study demonstrates that, when combined with the standardized measurement protocol, the ‘FlexYonio’ provides a reliable and practical instrument for assessing wrist extension with extended fingers. For the future we think the ‘FlexYonio’ may become more user friendly with use of lighter material, and integrating it with a digital goniometer, so the measurement can be done by one rater. Besides, we would like to support other centers who are interested in monitoring the FDP length in implementation of the ‘FlexYonio’ in their clinical practice.

## Conclusion

The newly developed ‘FlexYonio’ with its measurement protocol is a feasible, easy to use, and reliable instrument for monitoring shortening FDP by measuring the wrist extension with extended fingers. This instrument can help in early signaling of shortening of the FDP and timely initiating the use of preventive measures such as stretching the hands in prayers positioning or using hand orthoses.

## Supplemental Material

sj-docx-1-jnd-10.1177_22143602251362027 - Supplemental material for ‘FlexYonio’; a reliable instrument to support monitoring the length of the long finger flexors in Duchenne muscular dystrophy (DMD) Supplemental material, sj-docx-1-jnd-10.1177_22143602251362027 for ‘FlexYonio’; a reliable instrument to support monitoring the length of the long finger flexors in Duchenne muscular dystrophy (DMD) by Saskia LS Houwen- van Opstal, Maaike Pelsma, Yolanda MEM van den Elzen, Laura JB Merkenhof, Tamara P Popping- De Vries, Jan T Groothuis, Menno van der Holst and Edith HC Cup in Journal of Neuromuscular Diseases
