# Supplementary material for: ‘FlexYonio’; a reliable instrument to support monitoring the length of the long finger flexors in Duchenne muscular dystrophy (DMD)
Source: J Neuromuscul Dis. 2025 Aug 17;13(3):209–15. doi: 10.1177/22143602251362027 (PMC13438776; doi:10.1177/22143602251362027)
Supplement: Supplementary file 1 [file sj-docx-1-jnd-10.1177_22143602251362027.docx]

**Supplementary material**

**Measurement protocol**:

For this study the measurement with the “FlexYonio” will be repeated three times instead of one time (for the intra-reliability) and it will be executed in the beginning and additionally at the end (2x) (for the inter-reliability) of the physiotherapy- and occupational therapy assessment (total duration of 90 minutes) during the yearly control visit at Radboudumc. So in short, the measurement is performed 2x3 times, by two different therapists. The therapists report the outcome without showing this to each other. An independent researcher will collect the outcomes in a database.

Time scheme:

Start visit : T1 (3 measurements) ----------90 min assessment------------- T2(3 measurements), end visit

Before the ‘FlexYonio’ can be used, it is necessary to assess the mobility of the wrist and finger joints separately. If the extension of the fingers is limited, the FlexYonio cannot be used. If the wrist cannot be extended, the ‘FlexYonio’ should change position as is showed in figure 2b

*Starting position*: Examinator 1(E1) is standing next to the patient, examinator 2(E2) is in diagonally front of the patient. The patient is in sitting position with a 45 degrees flexed elbow, forearm in pronated position if this position is not possible, the possible starting position will be recorded. Both sides (left and right) are measured.

*Preparation*: First E1 determinates if the maximal passive wrist extension is from neutral position to dorsal extension, or limited between neutral position and palmar flexion. This starting position determinates the position of the hinge of the “flexYonio”, see picture 2 and 3.

Measurement: E1 positions the forearm of the patient on the longest side of the “flexYonio”, the other hand positions the finger joints on the shortest side of the “flexYonio”. In starting position no tension on the FDPs. From this position, move the shortest board to dorsal flexion until the end range of motion is reached. The end range of motion is reached when the tips of the fingers are bending, or when the researcher experiences a solid resistance. At this moment, E2 measures the angle of the two boards with a goniometer.

*Report*: The measured angle is reported of both sides as seen in table 1. If neutral position of the wrist is not feasible, the reported outcome will be negative. E2 reports the measurement without showing the result to E1. The second measurement, E1 and E2 roles are switched. An independent researcher collects all the results for analysis.
